# Electric Field Induced Electrorotation of 2D Perovskite Microplates

**DOI:** 10.3390/mi12101228

**Published:** 2021-10-09

**Authors:** Ruifu Zhou, Daobiao Hong, Siyu Gao, Yu Gu, Xuhai Liu

**Affiliations:** 1 College of Microtechnology & Microtechnology, Qingdao University, Qingdao 266071, China; 2020020607@qdu.edu.cn; 2 College of Materials Science and Engineering, Nanjing University of Science and Technology, Nanjing 210094, China; daobiaohong@mail.sim.ac.cn; 3 Materials Science and Engineering Department, Carnegie Mellon University, Pittsburgh, PA 15219, USA; siyugao@andrew.cmu.edu

**Keywords:** perovskites, nanomotor, AC voltage, contactless measurement, nanosheet

## Abstract

High precision-controlled movement of microscale devices is crucial to obtain advanced miniaturized motors. In this work, we report a high-speed rotating micromotor based on two-dimensional (2D) all-inorganic perovskite CsPbBr_3_ microplates controlled via alternating-current (AC) external electric field. Firstly, the device configuration with optimized electric field distribution has been determined via systematic physical simulation. Using this optimized biasing configuration, when an AC electric field is applied at the four-electrode system, the microplates suspended in the tetradecane solution rotate at a speed inversely proportional to AC frequency, with a maximum speed of 16.4 × 2π rad/s. Furthermore, the electrical conductivity of CsPbBr_3_ microplates has been determined in a contactless manner, which is approximately 10^−9^–10^−8^ S/m. Our work has extended the investigations on AC electric field-controlled micromotors from 1D to 2D scale, shedding new light on developing micromotors with new configuration.

## 1. Introduction

High precision-controlled movements of micro-objects are crucial for achieving advanced micro- and nanomotors [1,2,3], which might enable a plethora of various cutting-edge applications, such as drug delivery for in-vivo treatment [4,5], environmental remediation [6,7], biosensor and other bio-chemical implementations [8,9,10]. Current related research efforts have been mainly based on one-dimensional (1D) nanowires. For instance, as early as in 2005, a high rotation-speed micromotor prototype made of metal nanowires was applied to drive a dust particle into controllable circular motion [11]. More recently, an artificial nanomotor composed of two nickel-nanowire arms with a central gold-nanowire body has been demonstrated to propel twelve body lengths per second [12]. On the other hand, a multitude of manners have been developed to drive the nanowire-based motors via transferring external energy into mechanical motion, e.g., by chemical fuels [13,14], acoustic [15], magnetic [16,17], optic [18,19,20] and electric-induced external energy [21]. Among them, the electrically driven technique exhibits benefits that go further than being applicable for wide spectrum of nanowires, regardless of their metallic or non-metallic nature and also enabling simultaneous control of lateral alignment and synchronous rotation [22,23]. More importantly, by combining alternating and constant electric fields via four-electrode configuration, the targeted nanowires can be positioned at an arbitrary location in between the electrodes [24,25,26], as well as efficiently carrying out contactless conductivity measurements [27,28].

However, it is still a significant challenge to precisely control the nanowire-based micromotors, because the viscous force dominates the motion with nonnegligible Brownian motion in the nanometer scale with a very low Reynolds number [29,30]. This reinforces the necessity of developing miniaturized components in the micrometer scale (>10 µm), which is required by, for instance, a microrobot with size of several hundred micrometers [31,32]. From this perspective, two-dimensional (2D) microplates can be regarded as ideal candidates in composing the miniaturized motors in micrometer scale. For example, Enachi et al. developed a micromembrane consisting of TiO_2_ nanotube arrays, and the micromembrane with lateral size larger than 100 µm^2^ demonstrated an effective cargo loading and transport under UV stimuli [33]. Another planar “pancake-like” micromachine with diameter of 300 µm has been recently reported, in which light-controlled heartbeat-like pumping function was realized [34]. It should be clarified that “2D” here not only refers to a nanosheet with thickness less than 10 nm, but also include other planar configurations, as long as the lateral size is much larger than the thickness. Aside from the abovementioned 2D micromotors controlled by light stimuli, a diversity of other manners has been applied to power 2D micromotors, such as fuel-driven and magnetically driven techniques [35,36,37,38,39,40,41,42]. Nevertheless, the experimental work relating to 2D micromotors still lags its 1D-nanowire counterpart mainly because of two aspects, i.e., suitable 2D material and optimal precisely controlled technique.

In this work, we have systematically studied high-speed rotating micromotors based on all-inorganic perovskite CsPbBr_3_ microplates precisely controlled by external electric field, and further determined the electrical conductivity of CsPbBr_3_ microplate in a contactless manner. Firstly, in terms of the 2D material, CsPbBr_3_ microplates have been implemented in bewildering variety of different electrical and optoelectronic applications, owing to its large absorption coefficient, high photoluminescence quantum efficiency, ambipolar semiconductor characteristics, gradually improved environment stability [43,44,45,46,47,48]. Secondly, as for the external stimuli, inspired by the electric-driven nanowire micromotors and assisted by systematic physical simulations, we have applied the four-electrode configuration to precisely control the rotation speed of the 2D microplates, which reaches up to 16.4 × 2π rad/s. The electrical conductivity measured using this four-electrode system is approximately 10^−^^9^–10^−^^8^ S/m. Our work has extended the investigations on AC electric field-controlled micromotors from 1D to 2D scale, offering opportunities to develop micromotors with new configuration.

## 2. Materials and Methods

### 2.1. Synthesis of CsPbBr_3_ Microplates

Firstly, under the condition of stirring magnetically, we injected 0.2 mL precursor (1 mmol CsBr and 0.5 mmol PbBr_2_ dissolved in 15 mL DMSO) into 1 mL octadecylamine/acetic acid solution (0.05 g/mL). This is followed by the addition of 15 mL toluene into the solution with further magnetic stirring. Next, the chemical reaction was stopped by centrifugation at a speed of 5000 rpm for one minute. The precipitation was eventually redispersed in toluene and washed once again, and then dispersed in 4 mL of toluene.

### 2.2. Device Characterization

**Figure 1:**
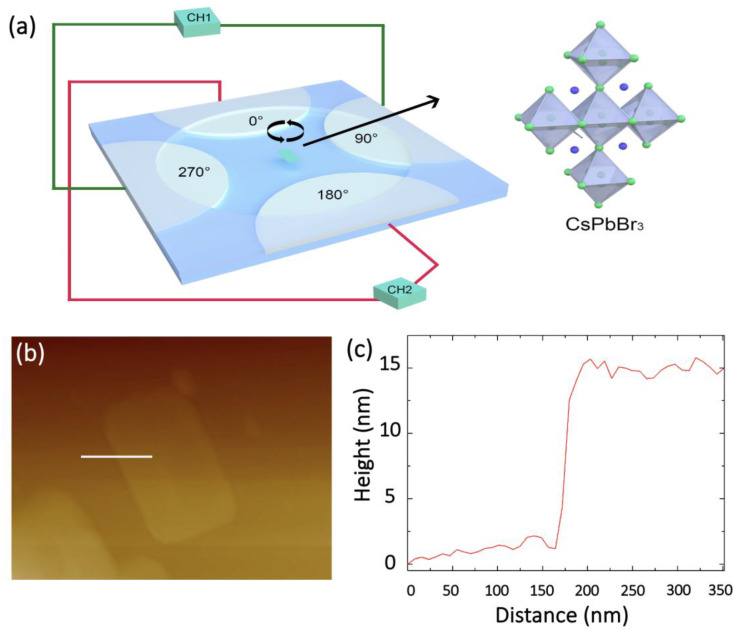
(**a**) Schematic illustration of the four-electrode configuration for biasing micromotor, with inset showing the crystalline structure of CsPbBr_3_. (**b**) AFM image of a typical single CsPbBr_3_ nanosheet. (**c**) Cross-sectional profile of the typical single CsPbBr_3_ nanosheet marked by the straight white line in (**b**).

The dynamic process of the CsPbBr_3_ microplates suspended in solution were observed via a Seiwa Optical Microscope coupled with a CCD imaging acquisition device. The alternating electrical field was applied by a RIGOL DG1022U function generator, coupled with a Agitek ATA-2082 High Voltage Amplifier. Regarding the setup of the four-electrode system, a 1 mm thick copper plate was firstly cut into rectangular copper strips of 15 mm × 1 mm, then a glass plate of 30 mm × 20 mm was used as the carrier and four copper strips were placed on the glass plate in pairs with 1.5 mm in between, as schematically illustrated in Figure 1a. Afterwards, epoxy resin was poured around the copper strips. A circular area with a diameter of 10 mm was left in the middle. This process was completed by curing for 60 s at 120 °C via a hotplate.

## 3. Results and Discussion

Figure 1a presents the schematic illustration of the four-electrode experimental configuration, with inset showing a portion of crystalline unit of CsPbBr_3_, in which the copper electrodes are partially covered by epoxy resin, with a glass as the substrate. The CsPbBr_3_ microplates dispersed in the solution of tetradecane are syringe-dropped in the center area surrounded by the four-metal electrodes. The dispersed single CsPbBr_3_ microplate is a pile of multilayer nanosheets with consistent lattice orientation rather than randomly ordered thin nanosheets [49]. Figure 1b demonstrates a typical single layer of CsPbBr_3_ nanosheet, from which the microplate can be constructed. It can be observed that the height of a typical CsPbBr_3_ nanosheet is approximately 15 nm, as shown in the cross-sectional profile in Figure 1c.

**Figure 2:**
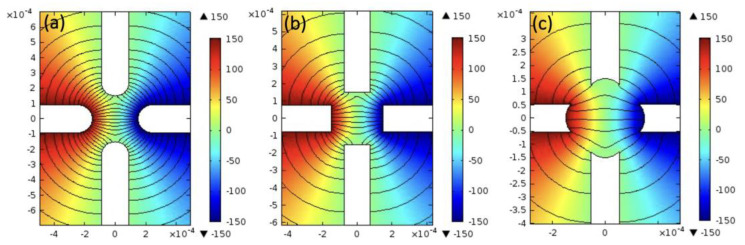
(**a**–**c**) Electric potential distribution of various electrode shapes, i.e., round electrodes, square electrodes and concaved electrodes. (length unit: m).

Firstly, to determine the optimized electrode configuration, we have systematically simulated the electric field distribution in the center of the orthogonal four-electrode system, both on 2D- and 3D-scale with the help of COMSOL Multiphysics 5.4. The system consists of four electrodes, which are connected to the external AC circuit, as shown in Figure 1a. In our simulations, we only simulate the field distribution when the AC circuit is just turned on, i.e., when *t* = 0 as described later in Equation (1). On the 2D scale, we have simulated different electrode shapes to optimize its electrical stability for the contactless conductivity measurements elaborated later in this work. The gradient of the electric field can greatly affect its stability. Therefore, a reasonable design of the electrode to reduce the gradient of the applied electric field without sacrificing the field strength is of great importance to the electrical characteristics of the microplates. Figure 2 compares the electric potential distribution of different electrode shapes, including round electrodes, square electrodes and concaved electrodes. It can be clearly observed that the round electrodes provide the optimized electric field distribution, i.e., a relatively strong and uniform electric field between the electrodes, as demonstrated in Figure 2a.

**Figure 3:**
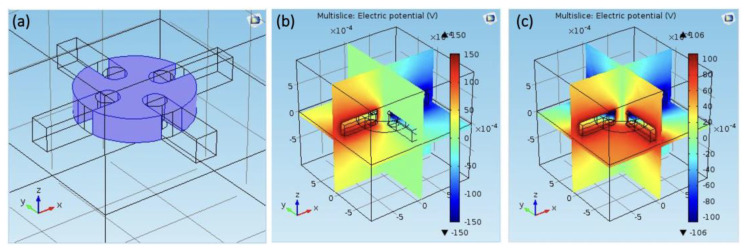
Electric potential distribution on 3D scale. (**a**) Geometry of the simulation electrodes. (**b,c**) Electrical potential distribution for two different voltage configurations with voltage applied across different electrodes (length unit: m).

On the 3D scale, we have simulated and measured the electric field stability at different depths to obtain the most suitable observation depth area. Figure 3 provides the geometric model on the 3D scale, as well as the electric potential distribution. Through the simulation, we can conclude that the most suitable observation depth ranges from 50 μm to 180 μm using round-shape electrodes, which have been selected as the actual biasing configuration in our experiments.

Regarding the operating mechanism, a rotating electric field can be generated by applying alternating-current (AC) voltage to the two sets of orthogonal metal electrodes with a *π*/2 phase difference, i.e.,

(1) $$\begin{array}{l} U_{1}=U_{0}\cos\omega t\\ U_{2}=U_{0}\cos(\omega t-\frac{\pi }{2}) \end{array}$$

where *U*_0_ is the amplitude and *ω* is the angular velocity. The electric field near the center exhibits a constant amplitude and rotates at the same angular velocity *ω*. The amplitude of the electric field *E*_0_ is related to *U*_0_ according to Equation (2) as follows:

(2) $$E_{0}=\alpha \frac{U_{0}}{d}$$

where *d* is the spacing between electrode and *α* is a correction factor, which is typically less than 1. This rotating electric field can exert a torque *T* on the microplates governed by the following equation [50]:

(3) $$T=-V\varepsilon_{2}E_{0}^{2}[\frac{(1-\tau_{1}/\tau_{2})\omega \tau_{1}}{1+{\left(\omega \tau_{1}\right)}^{2}}]$$

Here, *V* is the volume of the microplate. *τ*_1_ = *ε*_1_/*σ*_1_ and *τ*_2_ = *ε*_2_/*σ*_2_ are the characteristic relaxation times of the fluid and the microplate, respectively. *ε_i_* and *σ_i_* (*i* = 1, 2) represent the corresponding electric permittivity and conductivity, respectively. In this study, the fluid possesses a very low conductivity, i.e., σ_1_<< *σ*_2_ and its relaxation time is much larger than that of the microplate, i.e., *τ*_1_>> *τ*_2_. Based on this relation, Equation (3) can be simplified as:

(4) $$T=-V\varepsilon_{2}E_{0}^{2}\frac{{\left(\omega \tau_{1}\right)}^{2}}{1+{\left(\omega \tau_{1}\right)}^{2}}(\frac{1}{\omega \tau_{1}}-\frac{1}{\omega \tau_{2}})\approx VE_{0}^{2}\frac{\sigma_{2}}{\omega }$$

As indicated by Equation (4), the torque *T* is linearly proportional to the conductivity of the microplate. In the limit of low Reynolds number, the microplate suspended in the fluid can rotate at a constant velocity Ω in response to the torque:

(5) γΩ=T

where *γ* represents the rotational drag coefficient depending on the particle size and shape. For the microplate with much larger lateral size compared with its thickness, *γ* can be determined as [51,52]:

(6) $$\gamma =\frac{8\eta }{m\pi }V$$

where *η* is the viscosity of the fluid and *m* is the ratio of the thickness and lateral size of the microplate. Combining Equations (4)–(6), we can eventually obtain a relation governing the rotating velocity of the microplate, as presented in Equation (7) as follows:

(7) $$\Omega =\frac{m\pi }{8\eta \omega }\sigma_{2}E_{0}^{2}$$

**Figure 4:**
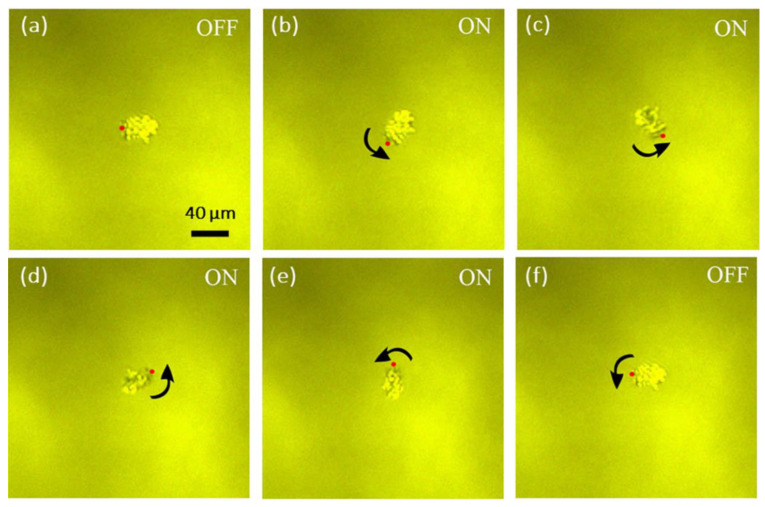
(**a**–**f**) Snap shots of rotating CsPbBr_3_ microplates in every 1/6 s.

Figure 4 demonstrates dynamic process of a rotating CsPbBr_3_ microplate driven by AC frequency of approximately 1.5 kHz. By applying AC voltages with different phase and frequencies in the range of 10 Hz to 100 kHz on the quadruple electrodes, a rotating AC electric field can be created to drive the CsPbBr_3_ microplate, as shown in Video S1 and Video S2 of Supplementary Materials. When a rotating AC electric field is applied to the four-electrode system, an electrical torque is imposed on the induced dipole moment of the CsPbBr_3_ microplates and force it to rotate. The rotation speed of the CsPbBr_3_ microplates can be precisely manipulated by adjusting the frequency of the AC electric field.

**Figure 5:**
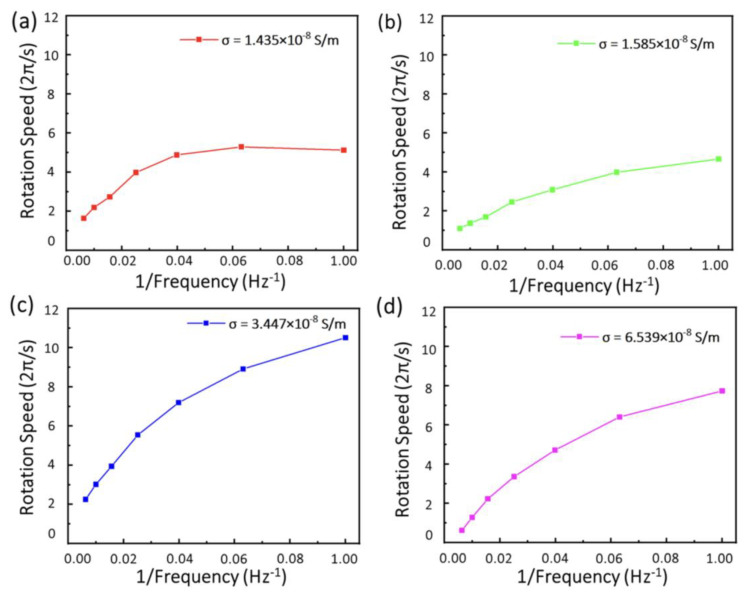
(**a**–**d**) Rotation speed dependent on AC frequency of the CsPbBr_3_ microplates with different sizes in the range from 690 µm^2^, 720 µm^2^, 1040 µm^2^ to 1470 µm^2^.

Due to influencing factors such as dielectric strain and electrolyte shielding effect, to minimize contact with the substrate to reduce the deviation in characterizing 2D material becomes increasingly important. Herein, we have obtained the specific conductivity of the CsPbBr_3_ microplates in a contactless manner. By processing the rotation of CsPbBr_3_ microplates through image processing, we can obtain the rotation speed of the CsPbBr_3_ microplates based on previously derived Equation (7). The conductivity of the CsPbBr_3_ microplates measured by this contactless manner ranges from 10^−^^9^–10^−^^8^ S/m. Moreover, as shown in Figure 5, the rotation speed of the CsPbBr_3_ microplates decreases with increasing the frequency of the AC electric field. The aforementioned theoretical derivation can be used not only for contactless measurement, but also for precise manipulation of microplates under an external AC electric field. This manipulation principle provides a feasible way to precisely control the rotation of 2D micromotors.

## 4. Conclusions

In summary, we applied AC electric field to precisely control all-inorganic perovskite CsPbBr_3_ microplates to rotate with high speed and further determined the electrical conductivity in a contactless manner. Moreover, the specific movement mechanism of the 2D material was systematically analyzed. In addition, the 2D and 3D simulation can confirm that the round electrode exhibits the optimized stability. Our work extended the investigations on AC electric field-controlled micromotors from 1D to 2D scale, shedding new light on developing micromotors with new configuration.

## Supplementary Materials

The following are available online at https://www.mdpi.com/article/10.3390/mi12101228/s1, Video S1: Dynamic rotation of a typical CsPbBr_3_ microplate at low AC frequency. Video S2: Dynamic rotation of a typical CsPbBr_3_ microplate at high frequency.
